# Diagnostic Confidence and Oral Cancer Screening: Insights From a Nationwide Cross-Sectional Study in Hungary

**DOI:** 10.1016/j.identj.2025.100878

**Published:** 2025-07-12

**Authors:** Peter Novák, Réka Magdolna Szabó, Gábor Braunitzer, István Vereb, Kinga Bágyi, Ákos Nagy, Árpád Joób-Fancsaly, Márk Ádám Antal

**Affiliations:** a Department of Operative and Esthetic Dentistry, Faculty of Dentistry, University of Szeged, Szeged, Hungary; b Department of Health Economics, Faculty of Medicine, University of Szeged, Szeged, Hungary; c dicomLAB Dental, Ltd, Szeged, Hungary; d Department of Operative Dentistry and Endodontics, Faculty of Dentistry, University of Debrecen, Debrecen, Hungary; e Department of Dentistry, Oral and Maxillofacial Surgery, University of Pecs, Pecs, Hungary; f Department of Oromaxillofacial Surgery and Stomatology, Semmelweis University, Budapest, Hungary

**Keywords:** Oral cancer, Screening practices, Knowledge, Oral health professionals, Self-confidence

## Abstract

**Objectives:**

Oral cancer screening is a critical preventive measure, yet various factors influence healthcare professionals’ willingness to engage in it. This nationwide cross-sectional study in Hungary examined the impact of diagnostic self-confidence, knowledge, and professional background on oral cancer screening and advisory behaviours among dentists, physicians, and clinical-grade medical and dental students.

**Methods:**

A questionnaire-based survey was conducted among 803 participants to assess their screening practices, knowledge, confidence, and educational needs.

**Results:**

Results indicated that diagnostic self-confidence was a key determinant of both screening and advisory behaviours, surpassing the impact of objective knowledge. Dentists and dental students demonstrated higher screening rates compared to physicians and medical students, reflecting differences in educational emphasis. While most respondents acknowledged gaps in their oral cancer knowledge, relatively few expressed a strong interest in further training. Online learning emerged as the preferred educational format, suggesting that the methods professionals favour may not be optimal for enhancing their screening and advisory abilities.

**Conclusions:**

These findings demonstrate the need for educational interventions that not only improve knowledge but also build confidence in early detection and patient communication. Future studies should explore training strategies that effectively bridge the gap between knowledge and practice, ensuring that healthcare professionals feel both prepared and motivated to engage in routine oral cancer screening and prevention efforts. Additionally, identifying barriers to participation in continuing education could help tailor learning opportunities that align with professional demands and time constraints. Understanding these factors is essential for optimizing oral cancer detection and reducing the burden of disease.

## Introduction

Regular screening tests and preventive counselling are effective tools for the prevention of cancers of the lip and oral cavity, as well as for reducing mortality, especially in high-risk populations.^1^, ^2^, ^3^ However, numerous obstacles prevent healthcare professionals at various levels of the healthcare system from performing these activities regularly. Dentists’ and physicians’ willingness to perform oral cancer screenings is shaped by interrelated factors, including perceived barriers such as time constraints and lack of equipment^4^; attitudes and beliefs about the efficacy of screening^5^^,^^6^; insufficient knowledge and training related to oral cancer^7^; social and professional norms^4^; patient awareness^8^; systemic challenges like the absence of formalized screening programs^6^^,^^9^; socio-economic barriers^10^; professional limitations^5^; cultural and demographic factors^11^; and technological challenges, including limited validation of diagnostic tools.^12^ Recent surveys have similarly reported low screening activity among dental professionals internationally.^13^

At the individual level, a professional’s training and knowledge about the disease are significant determinants of his willingness to conduct screenings and provide advisory services.^10^ Many practitioners feel inadequately prepared to identify early signs and symptoms of oral cancer, which can diminish their confidence and willingness to screen.^5^ Confidence appears to be a key factor: oral health professionals with greater confidence in their knowledge of oral cancer and in discussing oral health practices are more likely to perform screenings. This suggests that self-confidence in diagnostic abilities is a significant factor in the willingness to screen.^14^ Tax et al^15^ have also shown that possessing knowledge about oral cancer alone does not necessarily translate into action. This suggests that confidence may serve as a crucial link between knowledge and practice, but this relationship has not been often researched. This is an important question, as confidence being a key factor could have implications for selecting appropriate educational methods.

Hungary represents a compelling setting to investigate these issues. It has one of the highest incidence and mortality rates for oral cancer in Europe, second only to Slovakia, with an age-standardized incidence rate (ASR(W)) of 6.3 and a mortality rate of 2.9 per 100,000 population.^16^ Despite a levelling off in mortality trends over the last decade, Hungary remains a leader in oral cancer morbidity and mortality.^17^ The high rates of oral cancer in the country are primarily attributed to lifestyle risk factors such as tobacco and alcohol consumption,^18^ but increasing incidences among nonsmoking and nondrinking populations suggest additional, less understood determinants, which might well include systemic and psychological barriers to early detection. Hungary’s unique epidemiological profile makes it an ideal setting to explore factors influencing oral cancer screening behaviours, with the potential to generate findings applicable to other high-burden contexts.

In this study, we examined factors influencing the willingness of healthcare professionals and students in Hungary to perform oral cancer screenings and provide preventive advice as part of a broader national-level survey. This survey encompassed all clinical-grade students from medical and dental schools across the country, as well as practising medical doctors and dentists recruited through the Hungarian Medical Chamber. Through this extensive sample, the study aimed to explore systemic, educational, and psychological barriers to cancer screening and prevention within a high-burden context.

We hypothesized that diagnostic self-confidence and perceived sufficiency of knowledge would be key determinants of screening and advisory behaviours. Furthermore, we anticipated that demographic and professional factors, such as group affiliation and experience, would play significant roles in shaping these activities.

## Materials and methods

### Sampling

A cross-sectional questionnaire-based study was conducted among Hungarian dentists, physicians, dental students, and medical students. The survey was disseminated via two methods: emailed to all active members of the Hungarian Medical Chamber and manually distributed among clinical-grade students at Hungary’s four medical and dental universities. Clinical years are defined as years 4 to 6 for medical students and years 3 to 5 for dental students. Participation was voluntary and anonymous. Data collection will span from April 2022 to December 2023. At the time, the Chamber’s registry listed 49,683 medical doctors and dentists, with 650 dental students and 2571 medical students enrolled nationally. Notably, the registry does not distinguish between dentists and physicians due to overlapping qualifications in specialities such as maxillofacial surgery. Inclusion criteria were: active practitioners or clinical-grade students, native Hungarian speakers, and cognitive capacity to comprehend the study. Exclusion criteria were failure to meet any of these conditions. Informed consent was obtained, and forms were stored separately to maintain anonymity. The study was approved by the Hungarian Medical Research Council’s Scientific and Research Ethical Committee (Approval number: IV/6905-1/2021/EKU).

### Questionnaire design and content

The survey assessed participants’ knowledge, attitudes, practices, and perceived barriers related to oral cancer prevention and screening, including the impact of COVID-19. It comprised 18 questions (see Supplementary File), adapted from earlier studies^19^, ^20^, ^21^ or developed by our group. Questions adapted from earlier studies were translated according to published standardized procedures.^22^ The questionnaire covered demographic and professional background, routine screening practices, oral cancer knowledge, diagnostic confidence, referral practices, educational needs, and pandemic-related changes.

Administered in Hungarian, the survey was pilot-tested with 10 dentists and physicians of varying experience. Feedback on clarity, completion time, and content relevance was used to refine the final version.

The first section collected demographics: sex, age, profession (dentist, physician, dental/medical student), years of experience, specialization, and workplace setting (urban/rural). Screening practices were assessed with direct questions about routine oral mucosa examinations, including for high-risk patients.

Knowledge was measured through open-ended questions, such as listing primary oral cancer risk factors (Question 7) and clinical signs (Question 10), evaluated against standardized lists from textbooks.^23^, ^24^, ^25^, ^26^

Diagnostic confidence was assessed on a 4-point scale from ‘very confident’ to ‘very uncertain’. Referral preferences for suspected cases were recorded through multiple-choice questions. Self-perceived knowledge sufficiency and interest in further training were also captured, alongside preferred training formats (eg, online courses, workshops).

COVID-19’s impact on screening practices was assessed separately; these results are discussed elsewhere.

## Outcomes and data analysis

### Outcomes

The study focused on several key outcomes related to oral cancer diagnosis and prevention behaviours. Primary outcomes included regular screening and provision of preventive advice, both assessed as binary variables. Diagnostic self-confidence was evaluated on a four-level scale (‘very confident’ to ‘very uncertain’), while objective cancer knowledge was measured continuously, based on the number of correctly identified risk factors and clinical signs.

Educational needs were assessed by participants’ interest in further training and preferred training formats, including professional information packages, workshops, seminars, and online courses. Demographic data such as age, professional group affiliation, and experience were collected to contextualize the analysis.

### Data analysis

All analyses were conducted using Jamovi (version 2.3.28) and G*Power (version 3.1.9.7). Descriptive statistics summarized demographic characteristics and screening and advisory behaviours. Binomial logistic regression models assessed predictors of regular screening and preventive advice, including group affiliation, diagnostic confidence, self-perceived knowledge sufficiency, and experience. To harmonize students’ (grade-based) and practitioners’ (year-based) experience, a z-transformation was applied.

Posthoc power analyses confirmed sufficient sample size for reliable regression modelling. For regular screening, the event count (571/803) far exceeded the minimum recommended threshold (at least 10 events per predictor, a minimum of 80 events),^27^ yielding a calculated power of ∼98%. A similar power level was achieved for preventive advice outcomes.

To explore predictors of diagnostic self-confidence, a multinomial logistic regression was conducted, with self-perceived sufficiency, objective knowledge, and group affiliation as predictors. Objective knowledge was calculated by summing correctly identified risk factors and symptoms.

Because sex showed no significant effects and literature suggests it is less relevant in professional screening behaviours,^28^^,^^29^ it was excluded from the models to preserve statistical power.

## Results

### Demography

The sample included 803 respondents: 184 physicians (22.9%), 127 medical students (15.8%), 164 dentists (20.4%), and 328 dental students (40.8%). Response rates were 14.1% among students and 0.7% among practising professionals. Table 1 summarizes the demographic characteristics.

**Table 1:** Demographic characteristics of the groups.

	Age	Sex
D	39.5 (±13.2)	M: 84 (51.2%)
*N* = 164		F: 80 (48.8%)
DS	23.7 (±1.8)	M: 119 (36.3%)
*N* = 328		F: 209 (63.7%)
MD	46.6 (±14.3)	M: 84 (45.7%)
*N* = 184		F: 100 (54.3%)
MS	24.3(±3.05)	M: 32 (25.2%)
*N* = 127		F: 95 (74.8%)

Age is given in years (± SD), sex is shown as N (% within group).

D, dentist; DS, dental student; MD, physician; MS, medical student.

Dentists had a mean age of 39.5 years (±13.2) and physicians 46.6 years (±14.3), while dental and medical students averaged 23.7 (±1.8) and 24.3 (±3.05) years, respectively. Male-to-female ratios varied across groups but were relatively balanced (details in Table 1).

Among practitioners, dentists reported a median of 10.5 years in practice (range: 0-53), and physicians 19.0 years (range: 0-62). Most held at least one speciality certificate (dentists 62.8%, physicians 77.0%). Dominant dental specialities were restorative dentistry (27.0%), dento-alveolar surgery (25.5%), and dental/oral diseases (23.4%). In medicine, family practice (32.5%) and occupational medicine (12.4%) were the most common.

The majority of respondents (85.7%) worked or studied in urban settings (county seats or the capital).

### Cancer knowledge

Cancer knowledge was assessed by the number of correctly identified oral cancer signs and risk factors. Table 2 summarizes the results. Across all groups, respondents identified approximately three correct items in each category, with less than 10% failing to provide any correct answer.

**Table 2:** The number of correctly identified clinical signs and risk factors (mean, minimum-maximum), and the percentage of respondents who failed to provide a correct answer for each group.

	Correctly identified clinical signs	No correct identification
D (*N* = 164)	3.01 (0-8)	1.8%
DS (*N* = 328)	2.71 (0-7)	7.0%
MD (*N* = 184)	2.73 (0-8)	4.9%
MS (*N* = 127)	3.52 (0-7)	8.7%

	Correctly identified risk factors	No correct identification

D (*N* = 164)	3.22 (0-9)	3.7%
DS (*N* = 328)	2.80 (0-7)	6.4%
MD (*N* = 184)	2.48 (0-8)	7.1%
MS (*N* = 127)	2.81 (0-6)	3.9%

Qualitative analysis revealed similar patterns: ulceration, exo-/endophytic growth, and white lesions were the most commonly recognized clinical signs, each identified by over 50% of respondents in at least two groups. Among risk factors, smoking and alcohol use were the most frequently cited (70%-96% recognition), while other factors were less well known (3.6%-36%).

Detailed percentages for each risk factor and clinical sign are presented in Table 3.

**Table 3:** Risk factors and clinical signs associated with oral cancer as identified by the respondents.

Risk factor				
	D	DS	MD	MS
Smoking	96.34%	93.29%	93.48%	92.91%
Alcoholism	87.80%	81.10%	69.02%	80.31%
Chronic irritation	35.98%	20.43%	7.61%	5.51%
Poor oral hygiene	34.76%	18.90%	34.24%	32.28%
Viral infection	20.12%	25.00%	12.50%	30.71%
Genetic predisposition	11.59%	11.28%	13.59%	8.66%
Sunlight (UV radiation)	10.37%	8.23%	1.63%	3.15%
Hot, spicy food	6.71%	6.71%	4.89%	14.17%
Chemical agents	6.10%	4.57%	4.35%	3.94%
Immunocompromised states	4.88%	3.66%	3.26%	7.09%
Vitamin deficiency	3.66%	1.52%	1.09%	N/A
Fungal infection	2.44%	4.88%	1.09%	0.79%
Failed to provide acceptable response	3.66%	6.40%	7.07%	3.94%

Clinical sign				
	D	DS	MD	MS

Ulceration	75.00%	60.67%	66.30%	58.27%
Exo- or endophytic growth	69.51%	52.74%	55.98%	43.31%
White lesions	45.73%	58.23%	51.09%	40.16%
Red lesions	34.15%	39.63%	23.91%	27.56%
Bleeding	18.90%	17.99%	21.74%	25.98%
Other discoloration	17.68%	18.29%	17.39%	12.60%
Pain	17.68%	10.37%	16.30%	18.11%
Difficulty with swallowing	13.41%	5.79%	13.59%	16.54%
Lymph node enlargement	6.10%	5.18%	7.07%	N/A
Difficulty with speech	3.05%	2.13%	0.54%	7.87%
Failed to provide acceptable response	1.83%	7.01%	4.89%	8.66%

Percentages of respondents who identified the given item by group.

D, dentist; DS, dental student; MD, physician; MS, medical student.

### Screening and advisory activity

Screening and preventive behaviours are summarized in Table 4. Dentists (97.6%) and dental students (85.4%) reported the highest rates of regular oral cancer screening, while physicians (40.8%) and medical students (44.1%) reported lower rates. Preventive advice was provided less frequently across all groups, with dentists again leading (64.0%). A small proportion of respondents reported screening only high-risk patients: 1.3% of dentists, 6.6% of dental students, 11.8% of physicians, and 12.7% of medical students.

**Table 4:** The percentages of those dentists (D), dental students (DS), physicians (MD), and medical students (MS) who reported doing regular oral cancer screening and giving preventive advice regularly.

	Does screening as a routine	Gives preventive advice regularly
D (*N* = 164)	97.6%	64.0%
DS (*N* = 328)	85.4%	34.5%
MD (*N* = 184)	40.8%	31.5%
MS (*N* = 127)	44.1%	32.3%

Binomial logistic regression analyses identified professional group, diagnostic confidence, and self-perceived sufficiency of knowledge as significant predictors of regular screening (Table 5), explaining 32.8% of variance (*R*² = 0.328). Compared to physicians, dentists (OR = 48.2) and dental students (OR = 15.3) had substantially higher odds of regular screening. Higher diagnostic confidence and perceiving one’s knowledge as sufficient also significantly increased screening odds. Dentists had the highest levels of confidence and self-perceived knowledge sufficiency, followed by medical professionals and students (Supplementary Table S1).

**Table 5:** Results of the binomial logistic regression analysis for regular screening.

Predictor	Estimate	SE	*Z*	*P*	OR
Experience					
*z*-score	0.193	0.133	1.45	.147	1.213
Group					
DS – MD*	2.727	0.306	8.92	<.001	15.294
D-MD	3.875	0.552	7.02	<.001	48.198
MS-MD	0.509	0.321	1.59	.112	1.664
Diagnostic self-confidence					
Confident – Very uncertain*	1.948	0.398	4.89	<.001	7.016
Uncertain – Very uncertain	0.760	0.325	2.34	.019	2.138
Very confident – Very uncertain	1.859	0.686	2.71	.007	6.418
Self-perceived sufficiency of knowledge					
Yes – No*	1.063	0.290	3.67	<.001	2.896

D, dentist; DS, dental student; MD, physician; MS, medical student.

⁎ Indicates the reference category or level.

**Fig. 1:**
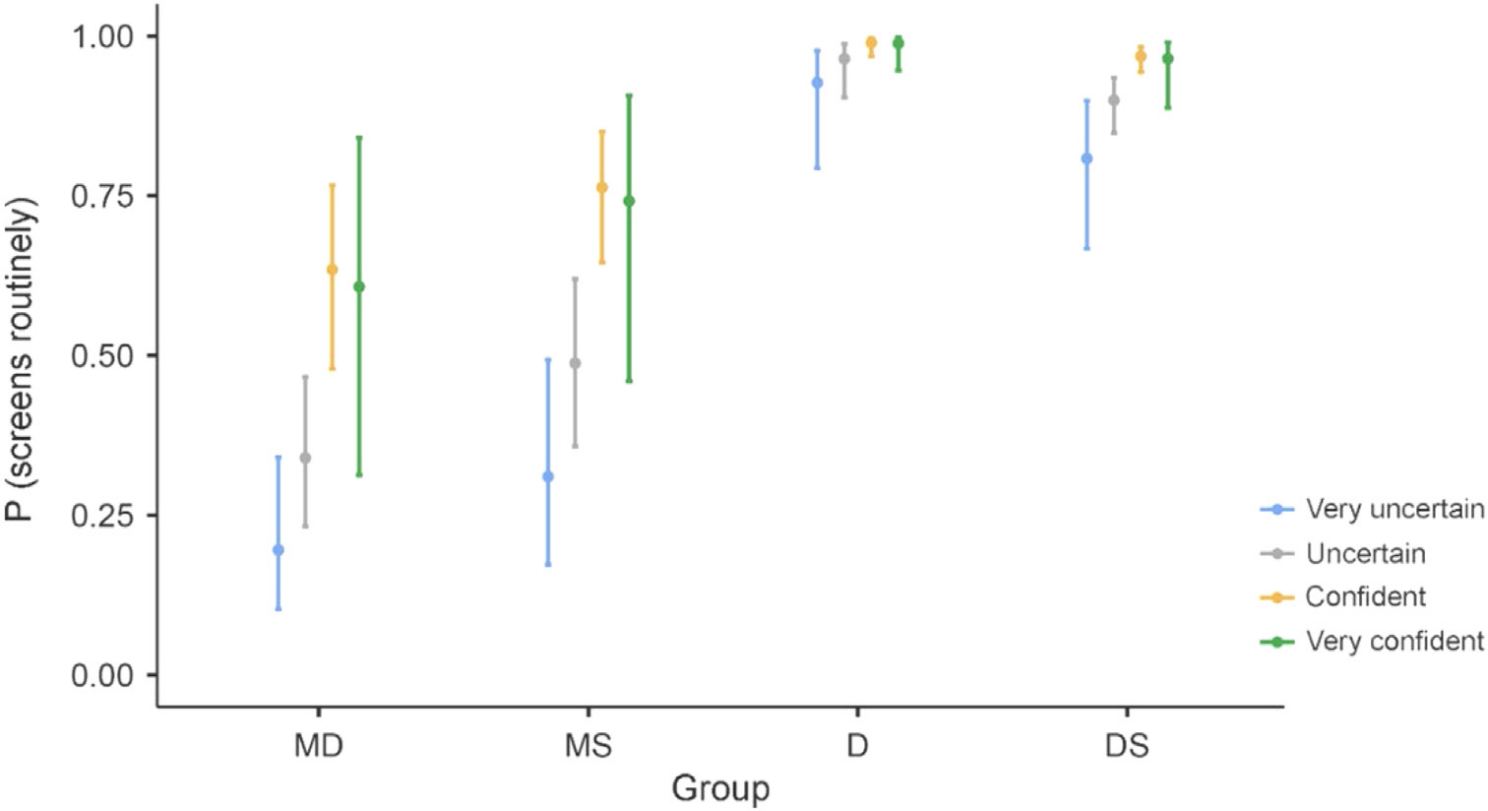
The probability of regular screening as determined by group affiliation and diagnostic self-confidence. D, dentist; DS, dental student; MD, physician; MS, medical student.

A graphical summary of screening probability by group and confidence level is shown in Figure 1.

Predictors of providing regular preventive advice were similar to those for regular screening. Binomial logistic regression identified experience, group affiliation, diagnostic confidence, self-perceived knowledge sufficiency, and regular screening behaviour as significant factors, with the model explaining 18.1% of the variance (*R*² = 0.181) (Supplementary Table S2).

Compared to physicians, dentists (OR = 2.63) and dental students (OR = 2.14) had significantly higher odds of offering preventive advice. Greater diagnostic confidence and perceiving one’s knowledge as sufficient were also associated with increased advisory activity. Experience showed a significant positive effect, unlike in the screening model. Medical students had higher odds of giving advice compared to physicians, but the difference did not reach statistical significance (Supplementary Table S2).

**Fig. 2:**
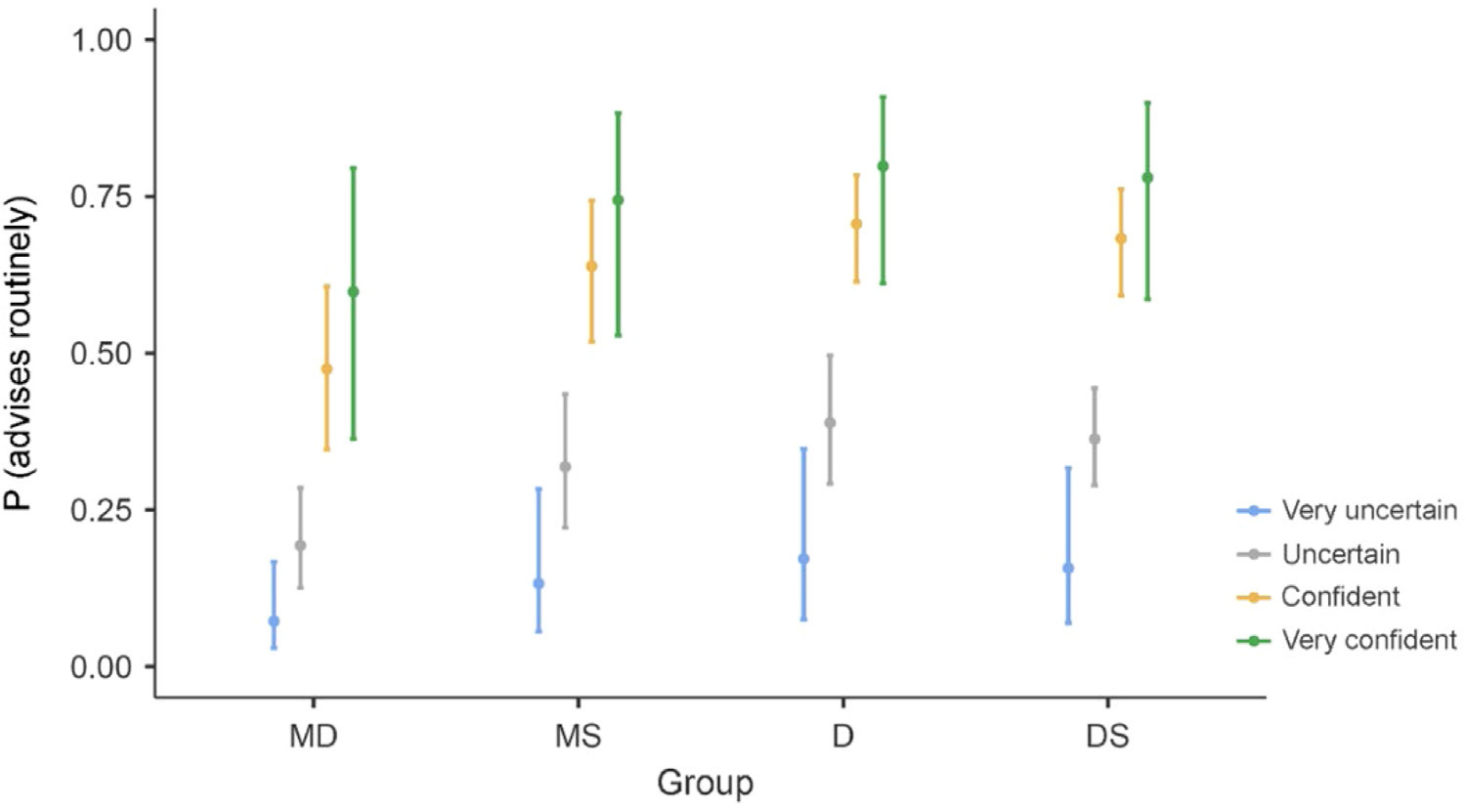
The probability of regular cancer prevention advisory activity as determined by group affiliation and diagnostic self-confidence. D, dentist; DS, dental student; MD, physician; MS, medical student.

Figure 2 illustrates the probability of regular preventive advice according to group affiliation and confidence level.

### Effects on diagnostic self-confidence

Multinomial logistic regression identified self-perceived sufficiency of knowledge as the strongest predictor of diagnostic confidence. Respondents who perceived their knowledge as sufficient were 4.93 times more likely to describe themselves as ‘uncertain’ rather than ‘very uncertain’, 35.28 times more likely to be ‘confident’, and 74.87 times more likely to be ‘very confident’ (all *P* < .001).

Objective knowledge also had a significant but smaller effect: each additional correct answer modestly increased the likelihood of higher confidence levels.

Group affiliation further influenced confidence. Dentists were significantly more likely than physicians to report higher confidence, particularly at the ‘confident’ and ‘very confident’ levels. Differences between physicians and students were smaller and often nonsignificant.

Detailed statistical results are provided in the Supplementary Material.

### Self-perceived need for cancer education and education preferences

Most participants perceived their oral cancer knowledge as insufficient, particularly among medical students (79.5%) and dental students (77.4%), followed by physicians (75.0%) and dentists (54.9%) (Supplementary Table S1). However, the proportion expressing interest in further education was much lower across all groups: only 12.1% of medical students, 18.3% of dentists, 18.8% of physicians, and 38.5% of dental students indicated a desire for additional training.

Online courses and information packs were the most preferred formats for further education across all groups, while in-person seminars and workshops were less favoured (Supplementary Table S3).

## Discussion

This nationwide study examined factors influencing oral cancer screening and preventive behaviours among Hungarian dentists, physicians, and clinical-grade students. The key finding is that diagnostic self-confidence, rather than objective knowledge, was the strongest predictor of both screening and advisory activities. Dentists and dental students reported higher confidence and greater screening rates compared to their medical counterparts, likely reflecting differences in educational emphasis.

While objective knowledge contributed modestly to confidence, self-perceived sufficiency of knowledge had a far stronger impact. This suggests that boosting professionals’ confidence may be more effective for enhancing screening behaviours than focusing solely on knowledge acquisition. These results align with prior studies but also highlight important differences based on methodology and sample characteristics.

Diagnostic self-confidence and perceived sufficiency of knowledge strongly predicted screening behaviour. Even moderate increases in confidence significantly raised the likelihood of routine screening and preventive advice; for example, ‘very confident’ respondents were 6.4 times more likely to screen regularly than those who felt ‘very uncertain’. This supports findings by Marino et al,^14^ who also linked self-reported confidence to higher screening rates among Australian oral health professionals, although their observed odds ratios and overall screening rates were notably lower than ours. The higher screening rate among Hungarian dentists (97.6%) may reflect national differences in clinical expectations or training.

Our finding that perceived sufficiency had a stronger effect on confidence than objective knowledge aligns with the results of Hassona et al,^30^ who reported modest correlations between knowledge and diagnostic ability among Jordanian primary care providers. However, by using multinomial logistic regression, we were able to more precisely separate the effects of factual knowledge and self-perception, suggesting that subjective confidence plays a substantially greater role than objective knowledge in driving behaviour.

Tax et al^15^ also found a disconnect between knowledge and practice among dental hygienists, contrasting with our high screening rates among dentists and dental students. In our sample, 97.6% of dentists and 85.4% of dental students reported regular screening, compared to 40.8% of physicians and 44.1% of medical students. Preventive advice was less consistently offered, but dentists still led at 64%. Diagnostic confidence followed a similar pattern: 67.7% of dentists described themselves as ‘confident’ or ‘very confident’, compared to 21.0% to 31.5% in other groups.

These findings align with Langton et al,^31^ who reported that dentists were more likely than physicians to detect oral cancers early, often during asymptomatic visits. Although that study did not quantify confidence directly, the qualitative evidence supports our conclusion that perceived competence plays a central role in motivating screening and advisory behaviours.

Professional experience did not significantly predict screening activity, but it did influence the likelihood of providing preventive advice. This distinction mirrors findings by Marino et al,^14^ who observed that screening behaviour was more strongly associated with confidence and communication skills than with years of experience. It suggests that while screening can be protocol-driven, effective advisory activity may require more developed interpersonal skills, which often improve with time. However, this hypothesis could not be directly tested within the scope of our study.

Beyond the central role of confidence, other patterns have emerged. Although most participants, especially students, perceived their oral cancer knowledge as insufficient, relatively few expressed interest in further education. Leuci et al^32^ observed a similar trend among Italian dental hygienists, citing time constraints, reliance on supervising dentists, and a mismatch between perceived and actual knowledge as potential barriers. Although our study did not explore these factors directly, they likely contributed to the low demand for continuing education in our sample. For instance, Trifunovic-Koenig et al^33^ reported that heavy workloads were a key obstacle to participation in infection prevention training – suggesting that limited time may also discourage engagement in oral cancer education.

Respondents’ preference for online courses and information packs likely reflects time constraints and the demand for flexible learning formats, a trend accelerated by the COVID-19 pandemic. While online education offers accessibility, it may not be the most effective approach for building the practical skills and confidence necessary for real-world screening and advisory activities.

The literature supports this concern. Tax et al^15^ emphasized that knowledge alone is insufficient to change behaviour. Continuing education programs that incorporate hands-on training, gamification, or confidence-based learning have shown greater success in improving diagnostic skills and screening rates.^34^^,^^35^ Strategies such as role-playing, standardized patients, and communication-focused workshops also enhance practical abilities, although their effectiveness varies depending on delivery format and participant population.^36^, ^37^, ^38^, ^39^, ^40^

### Limitations and suggestions for future work

This study has several limitations. First, the response rate among practising professionals was very low (0.7%), particularly among physicians, which limits the representativeness of the sample and raises concerns about nonresponse bias. Although the overall sample size was sufficient for statistical analyses (even larger than that of similar studies^14^^,^^15^^,^^30^), future studies should aim to increase participation through incentives, institutional partnerships, or mixed-method approaches.

Second, the reliance on self-reported data introduces potential biases such as social desirability and recall errors. Objective assessments, such as case simulations or direct observations, would enhance data validity in future research.

Third, while many respondents acknowledged insufficient knowledge, few expressed interest in further education. Qualitative studies are needed to explore motivational and systemic barriers to participation in continuing education. In future studies, incorporating open-ended follow-up items in addition to multiple-choice questions may provide richer qualitative data and deeper insights into participant motivations and perceptions. This adjustment would help capture nuances that fixed-response formats might overlook, particularly in understanding diagnostic behaviour and educational needs.

Fourth, diagnostic confidence was measured subjectively. Combining self-assessments with external evaluations would provide a more accurate picture of competence.

Finally, the study’s findings are based on data from Hungary, a country with high oral cancer incidence and mortality. Replicating the research in different healthcare settings would help determine the broader applicability of these results.

## Conclusions

This study highlights diagnostic self-confidence as a key driver of oral cancer screening and preventive advice among healthcare professionals and students, surpassing the influence of factual knowledge. Although many participants recognized gaps in their knowledge, few expressed interest in further training, with online courses emerging as the preferred format.

However, reliance on online learning may not sufficiently build the confidence and practical skills necessary for effective screening behaviours. Future educational interventions should aim not only to improve knowledge but also to strengthen diagnostic confidence by using interactive and practical training methods.

## Ethics statement

This study conformed to the Declaration of Helsinki Ethical Principles for Medical Research Involving ‘Human Subjects’, adopted by the 18th World Medical Assembly, Helsinki, Finland, June 1964, as amended by the 64th World Medical Assembly, Fortaleza, Brazil, October 2013. The protocol was approved by the Hungarian Medical Research Council’s Scientific and Research Ethical Committee (Approval number: IV/6905-1/2021/EKU).

## Informed consent statement

Informed consent was obtained from all subjects involved in the study.

## Conflict of interest

There is no conflict of interest.

## Appendix Supplementary materials

Image, application 1

Image, application 2

## Footnotes

Supplementary material associated with this article can be found in the online version at doi:10.1016/j.identj.2025.100878.
