# Supplementary material for: Diagnostic Confidence and Oral Cancer Screening: Insights From a Nationwide Cross-Sectional Study in Hungary
Source: Int Dent J. 2025 Jul 12;75(5):100878. doi: 10.1016/j.identj.2025.100878 (PMC12275046; doi:10.1016/j.identj.2025.100878)
Supplement: Supplementary file 1 [file mmc1.pdf]

SURVEY ON AWARENESS OF HEAD AND NECK CANCERS AMONG PHYSICIANS, DENTISTS, DENTAL AND MEDICAL STUDENTS

NOTE: The English version is for illustrative purposes only. The language of the actual study questionnaire is Hungarian.

This questionnaire is designed to assess your knowledge, habits, and practices related to oral precancerous and malignant conditions. It also includes questions about your standard procedures and preventive measures in this area. The questionnaire is anonymous; please do not include any personally identifiable information. If you have any comments or feedback, feel free to write them on the back of the questionnaire.

1. **Your sex:**
   - ☐ Female
   - ☐ Male

2. **Your age:** ……….. (years)

3. **You are**
   - ☐ Physician (Years in practice:…….(years)
     If you are a specialist, your field:……………………………..
   - ☐ Dentist (Years in practice…….(years)
     If you are a specialist, your field:……………………………..
   - ☐ Medical student (Current year of study……..)
   - ☐ Dental student (Current year of study……..)

4. **Where do you currently work/study ?**
   - ☐ Capital city
   - ☐ County seat
   - ☐ Other city
   - ☐ Village

5. **Do you routinely examine the oral mucosa of your patients?**
   - ☐ YES
   - ☐ NO

6. **If you answered NO to the previous question, do you examine the oral mucosa of high-risk patients?**
   - ☐ YES
   - ☐ NO

7. **What do you consider to be risk factors for oral cancer?** (Please specify)

………

………

8. Do you regularly provide advice to patients about the risk factors of oral cancer

- ☐ YES
- ☐ NO

9. How would you assess your confidence regarding the clinical presentation and diagnosis of oral cancer?

- ☐ Very confident
- ☐ Confident
- ☐ Uncertain
- ☐ Very uncertain

10. What types of oral changes are associated with oral cancer? (Please specify)

………

………

11. If you suspect oral cancer in a patient, which specialist would you refer them to?

- ☐ Plastic surgeon
- ☐ ENT specialist
- ☐ Maxillofacial surgeon
- ☐ Oral medicine specialist
- ☐ Dental specialist
- ☐ General practitioner
- ☐ other: ....

12. Do you think you have sufficient knowledge regarding the prevention and detection of oral cancers?

- ☐ YES
- ☐ NO

13. Would you like to receive further information or training on oral cancer?

- ☐ YES
- ☐ NO

14. If yes, which format would you prefer? (Assuming all are available)

- ☐ Professional information package
- ☐ Short, daytime training

- ☐ Evening training
- ☐ Full-day seminar
- ☐ Online training

15. Since the beginning of the COVID-19 pandemic, oral cancer screenings and specialist consultations have become less accessible to patients.

Strongly disagree 1 2 3 4 5 Strongly agree

16. Since the beginning of the COVID-19 pandemic, patients have participated less in screening examinations.

Strongly disagree 1 2 3 4 5 Strongly agree

17. Since the beginning of the COVID-19 pandemic, patients have attended fewer medical or dental examinations and treatments.

Strongly disagree 1 2 3 4 5 Strongly agree

18. Since the resumption of primary care and specialist consultations, I have encountered more cases of cancer or precancerous conditions than before.

Strongly disagree 1 2 3 4 5 Strongly agree

Thank you for your cooperation!
