# Supplementary material for: Diagnostic Confidence and Oral Cancer Screening: Insights From a Nationwide Cross-Sectional Study in Hungary
Source: Int Dent J. 2025 Jul 12;75(5):100878. doi: 10.1016/j.identj.2025.100878 (PMC12275046; doi:10.1016/j.identj.2025.100878)
Supplement: Supplementary file 2 [file mmc2.docx]

SUPPLEMENTARY TABLES TO **DIAGNOSTIC CONFIDENCE AND ORAL CANCER SCREENING: INSIGHTS FROM A NATIONWIDE CROSS-SECTIONAL STUDY IN HUNGARY** BY NOVÁK ET AL.

**Table S1.** Descriptive statistics for the level of diagnostic self-confidence and self-perceived sufficiency of own cancer knowledge. D: dentist, DS: dental student, MD: physician, MS: medical student. Within-group percentages.

| **Level of diagnostic self-confidence** | | | | |
| --- | --- | --- | --- | --- |
|  | **D (N=164)** | **DS (N=328)** | **MD (N=184)** | **MS (N=127)** |
| Very confident | 8.5% | 1.2% | 5.4% | 3.1% |
| Confident | 59.1% | 19.8% | 26.1% | 26.0% |
| Uncertain | 30.5% | 72.9% | 53.8% | 56.7% |
| Very uncertain | 1.8% | 6.1% | 14.7% | 14.2% |
| Confident and very confident (sum) | 67.7% | 21.0% | 31.5% | 29.1% |
| Uncertain and very uncertain (sum) | 32.3% | 79.0% | 68.5% | 70.9% |
| **Perceives own cancer knowledge as sufficient** | | | | |
| yes | 45.1% | 22.6% | 25.0% | 20.5% |
| no | 54.9% | 77.4% | 75.0% | 79.5% |

**Table S2.** Results of the binomial logistic regression analysis for offering preventive advice on a regular basis. Asterisk (*) indicates the reference category or level. D: dentist, DS: dental student, MD: physician, MS: medical student.

| **Predictor** | **Estimate** | **SE** | **Z** | **p** | **OR** |
| --- | --- | --- | --- | --- | --- |
| **Experience** | | | | | |
| z-score | 0.292 | 0.111 | 2.64 | 0.008 | 1.3394 |
| **Group** | | | | | |
| DS-MD* | 0.763 | 0.285 | 2.68 | 0.007 | 2.1444 |
| D-MD | 0.965 | 0.276 | 3.49 | < .001 | 2.6255 |
| MS-MD | 0.579 | 0.327 | 1.77 | 0.077 | 1.7849 |
| **Diagnostic self-confidence** | | | | | |
| Confident – Very uncertain* | 2.435 | 0.465 | 5.24 | < .001 | 11.4139 |
| Uncertain – Very uncertain | 1.111 | 0.446 | 2.49 | 0.013 | 3.0387 |
| Very confident – Very uncertain | 2.918 | 0.627 | 4.65 | < .001 | 18.5103 |
| **Self-perceived sufficiency of knowledge** | | | | | |
| Yes-No* | 0.662 | 0.195 | 3.40 | < .001 | 1.9392 |

*indicates the reference category

**Table S3.** Preferred forms of further education regarding oral cancer across the groups. D: dentist, DS: dental student, MD: physician, MS: medical student.

| **Format** |  | **D** | **DS** | **MD** | **MS** |
| --- | --- | --- | --- | --- | --- |
| an online course | | 67.68 | 71.34 | 67.93 | 63.78 |
| information pack | | 34.15 | 51.83 | 40.22 | 47.24 |
| lunchtime meeting | | 23.17 | 28.05 | 27.17 | 33.07 |
| evening seminar | | 5.49 | 6.71 | 11.96 | 14.17 |
| a whole-day seminar | | 10.37 | 10.06 | 9.24 | 4.72 |
